# Revealing induced pluripotent stem cells' potential as a better alternative to embryonic stem cells for Parkinson's disease treatment based on single-cell RNA-seq

**DOI:** 10.1590/1414-431X2024e13482

**Published:** 2024-12-13

**Authors:** Sen Zhang, Xing Jiang, Min Yan, Zixiao Cheng, Jun Bi, Qinglu Wang, Ying Luo, Xuewen Tian

**Affiliations:** 1 Shandong Sport University, Jinan, Shandong Province, China; 2 Gdansk University of Physical Education and Sport, Gdansk, Poland; 3 Department of Clinical Laboratory, Zibo Central Hospital, Zibo, China

**Keywords:** ESCs, iPSCs, Dopamine neurons, Astrocytes, Single-cell RNA-seq

## Abstract

Both embryonic stem cells (ESCs) and the successful reprogramming of induced pluripotent stem cells (iPSCs) offer an unprecedented therapeutic potential for Parkinson's disease (PD), allowing for the replacement of depleted neurons in PD-affected brain regions, thereby achieving therapeutic goals. This study explored the differences in cell types between iPSCs and ESCs in the PD brain to provide a feasible theoretical basis for the improved use of iPSCs as a replacement for ESCs in treating PD. Signal cell RNA sequencing data and microarray data of ESCs and iPSCs were collected from the GEO database. scRNA-seq data were subjected to quality control, clustering, and identification using the Seurat R package to determine cell types and proportions in ESCs and iPSCs. Differential expression analysis was performed to identify differentially expressed genes between ESCs and iPSCs, and PPI network analysis was conducted using String. Based on scRNA-seq data, we identified 13 cell clusters in ESCs and 13 cell clusters in iPSCs. iPSCs were predominantly composed of immune cells and lacked astrocytes, neurons, and dopamine neurons compared to ESCs. iPSCs also exhibited lower cell type diversity compared to ESCs. At the gene level, iPSCs lacked key genes, such as *TH* and *GAP43* for nerve growth and development. At the metabolic level, the difference between ESCs and iPSC was mainly reflected in nerve cells and was closely related to the tumor-proliferation signature. iPSCs can be promoted to differentiate into cell types closer to or even replace ESCs, providing a better therapeutic option for PD treatment.

## Introduction

Parkinson's disease (PD) is the second most common neurodegenerative disorder after Alzheimer's disease, affecting over 6 million people worldwide, predominantly those over the age of 65. PD is characterized by symptoms including motor disorders, cognitive impairments, and mood disturbances, severely impacting the physical and mental well-being of patients (1). The pathological features of PD are mainly described as the degeneration of neurons in the substantia nigra pars compacta (SNpc) and ventral tegmental area (VTA), leading to dopamine (DA) depletion and the aggregation of α-synuclein (α-Syn) in the form of Lewy bodies, resulting in a series of symptoms (2,3). Currently, the primary treatment for PD remains DA replacement therapy, which uses levodopa as a direct precursor for DA synthesis to replenish DA levels in the PD-affected brain. However, this gold standard treatment can lead to diminished effectiveness and side effects such as dyskinesia (in 80% of patients) with continued use (4). Therefore, current research is actively seeking more effective approaches to treat PD.

Recent studies have proposed the transplantation of embryonic stem cells (ESCs) as a therapeutic strategy, demonstrating its great feasibility. The main principle is to leverage the ability of ESCs to differentiate into various cell phenotypes, along with their excellent proliferation and migration capabilities, to replace damaged brain cells in the PD environment and restore the impaired nigrostriatal pathway (5,6). Currently, this therapeutic approach has been validated by a substantial amount of experimental data. Whether in terms of morphology, molecular biology, or behavioral and neuroimaging studies, ESCs have been shown to reinnervate the nigrostriatal pathway in PD and achieve significant recovery of motor symptoms (7-10). Additionally, animal studies and clinical trials using fetal ventral mesencephalon (fVM) tissue have demonstrated that transplanted DA neurons can survive, integrate, and release into the striatum, thereby repairing abnormal cortical function and motor symptoms in PD patients (11,12). However, two potential limitations hinder the optimal application of ESCs as a therapeutic agent. Firstly, the generation of ESCs requires the destruction of donated fertilized eggs or early-stage embryos, raising significant ethical and legal concerns (13). Secondly, most ESC-derived cell transplants are allogeneic to the recipient patients, necessitating the use of immunosuppression protocols (14).

Based on this treatment strategy, the emergence of induced pluripotent stem cells (iPSC) technology has provided unprecedented possibilities for this approach, enabling the generation of disease- and patient-specific stem cells without damaging early embryos, and eliminating the need for immunosuppressive regimens. It has been widely studied and considered a new and effective strategy. Since 2007, when Takahashi et al. (15). discovered that a few transcription factors could reprogram cell differentiation, iPSCs have been extensively used in the study of PD-related neurodegenerative diseases to guide the fate of patient-specific cells. Although the scope is still limited, iPSCs are currently the most reliable and phenotypically similar models for PD (16). In addition, the FDA (Food and Drug Administration) has recently approved the application of iPSCs in humans without the requirement for animal testing, which again highlights the importance of iPSCs for PD treatment (17). However, iPSC-based cell therapies are not yet fully mature, and there is widespread variability in the differentiation potential between individual iPSCs. Therefore, this study used bioinformatics based on single-cell RNA sequencing data to compare and analyze the main cell types and quantities transformed by ESC treatment and iPSC treatment in the PD environment. The interactions between cell populations as well as whether iPSCs can achieve therapeutic effects similar to or exceeding ESCs under the induction of certain primers were also investigated.

## Material and Methods

### RNA sequencing data processing

**Figure 1:**
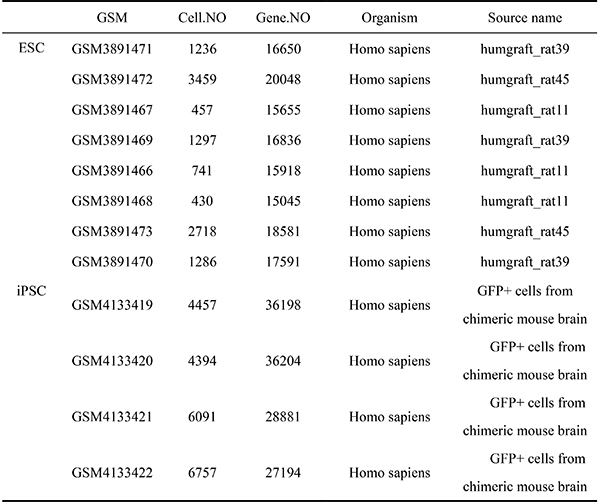
Dataset derived from the GEO (Gene Expression Omnibus) database (https://www.ncbi.nlm.nih.gov/geo/). Embryonic stem cells (ESCs) data were submitted on July 14, 2019 and updated on May 31, 2020, with a source name of “humgraft_rat39”. Induced pluripotent stem cells (iPSC) data were submitted on March 14, 2013, and updated on May 15, 2019, with a source name of “GFP+ cells from chimeric mouse brain”. Both datasets have a sample type of SRA, human organ, and data type of 10x genomics.

In this study, we utilized the Gene Expression Omnibus (GEO) database (https://www.ncbi.nlm.nih.gov/geo/) to collect publicly available high-throughput gene expression data. Thorough searches were performed on all datasets related to ESC and iPSC treatment strategies for PD, resulting in the selection of 12 microarray datasets (Figure 1). Among them, there were 8 microarray datasets (GSM3891471, GSM3891472, GSM3891467, GSM3891469, GSM3891466, GSM3891468, GSM3891473, GSM3891470) associated with the ESC treatment strategy. For the iPSC treatment strategy, there were 4 microarray datasets (GSM4133419, GSM4133420, GSM4133421, GSM4133422).

Firstly, the Seurat package in RStudio software was used to perform expression quantification on these single-cell sequencing data. Cell filtering was conducted based on the number of RNA molecules per cell (ranging from 200 to 2500) and mitochondrial content (setting the threshold of mitochondrial gene expression proportion in a single cell to 10%). The Harmony method was carried out to remove batch effects between samples. Next, highly variable genes (top 2000) were selected based on their level of variability.

### Identification and characterization of cell subtypes

After data filtering, the selected data were subjected to principal component analysis (PCA) (P<0.01). The UMAP dimension reduction method was then applied for visualization (using 10 principal components and a resolution of 0.5). Cell clustering and sub-clustering were performed using the clustering algorithm in the Seurat package. Based on existing research and sample characteristics, the cell subtypes in the samples were estimated. Known gene markers corresponding to each cell subtype were searched, and subtypes were classified and named based on these known gene markers. Cell subtypes that could not be defined were labeled as undefined cell types. Finally, bar plots were generated to visualize the proportions of different cell types in each group.

### Network construction

The marker genes from ESCs and iPSC single-cell data were analyzed using STRING (https://string-db.org/), and a network interaction plot between ESC and iPSC was constructed using the Cytoscape software.

## Results

### ESCs and iPSC scRNA-seq data analyses

**Figure 2:**
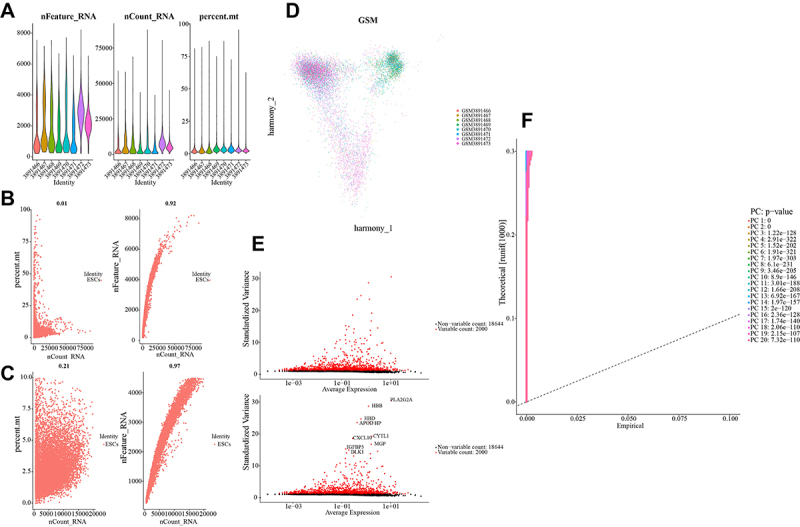
A, RNA counts obtained after quality control of embryonic stem cells (ESCs). **B** and **C**, Coefficient of variation of RNA after quality control in ESC cells. **D**, Batch-corrected results (top 2000) using the harmony method on the filtered data. **E**, Highly variable genes (HVGs) in ESC cells after batch correction, with the top 10 differentially expressed genes labeled. **F**, Principal component analysis (PCA) of the data results, determining the significant number of principal component (PC) groups from high-variance genes to high-variance gene clusters.

**Figure 3:**
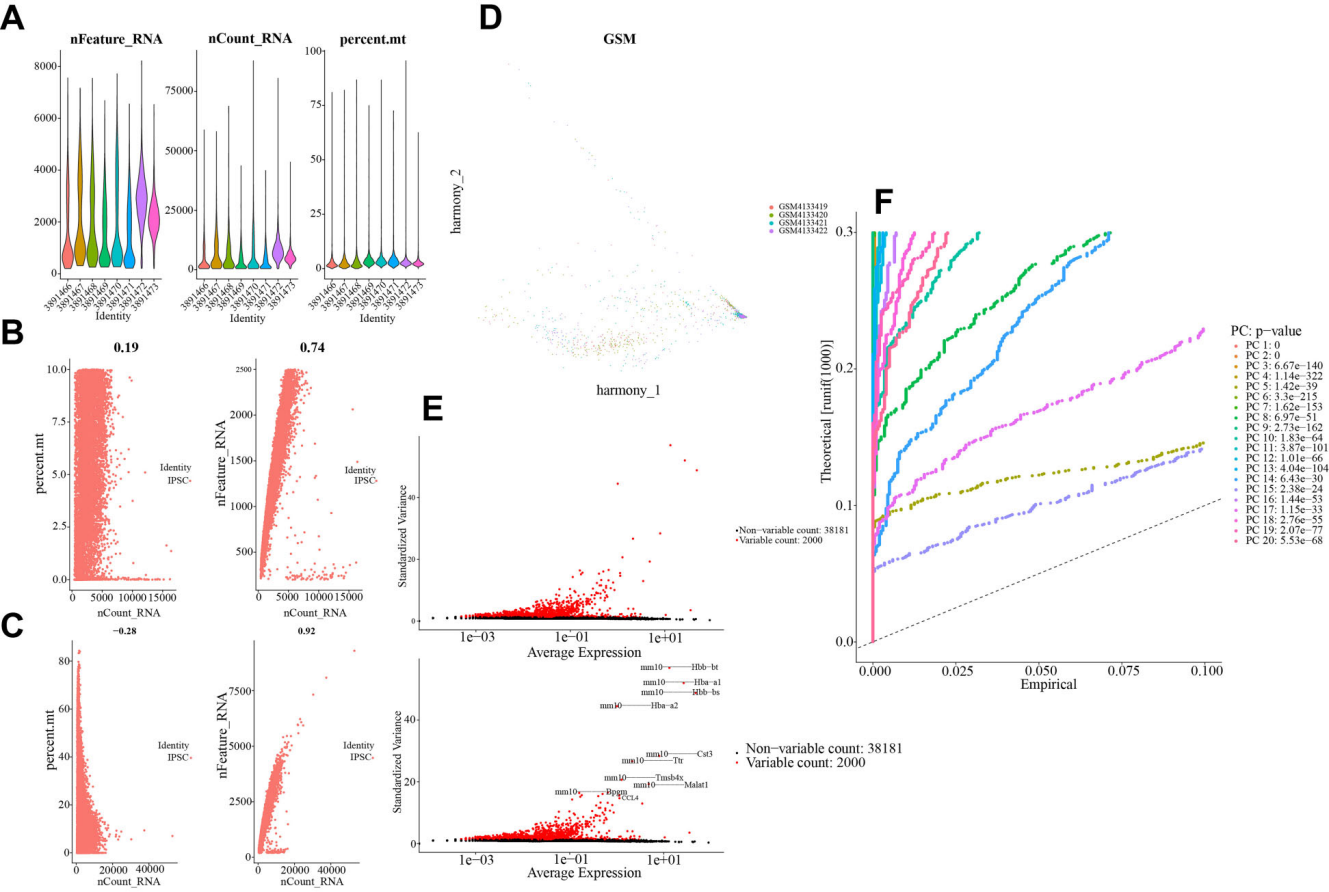
A, RNA counts obtained after quality control of induced pluripotent stem cells (iPSC) cells. **B** and **C**, Coefficient of variation of RNA after quality control in iPSC cells. **D**, Batch-corrected results (top 2000) using the harmony method on the filtered data. **E**, Highly variable genes (HVGs) in iPSC cells after batch correction, with the top 10 differentially expressed genes labeled. **F**, Principal component analysis (PCA) analysis of the data results, determining the significant number of principal component (PC) groups from high-variance genes to high-variance gene clusters.

In the ESCs treatment strategy, a total of 11,624 cells were obtained from 8 samples and in the iPSC treatment strategy, a total of 26,664 cells were obtained from 4 samples. After the initial step of cell quality control, we obtained the gene count (nFeature_RNA), sequencing depth (nCount_RNA), and percentage of mitochondrial genes (percent.mt) in the samples (Figure 2A-C and 3A-C). After filtering the data based on the subset of highly variable genes, batch effects were removed using the harmony method, and the merged batch-corrected PCA analysis plot was obtained (Figure 2D and 3D). After batch removal, magnet genes were further identified, and the top 2000 highly variable genes were obtained (Figure 2E and 3E). Finally, the significant number of PC clusters was determined to be 18 for ESC and 10 for iPSC based on the number of PCA principal components (Figure 2F and 3F), representing groups of highly variable genes.

### Differences between iPSC and ESC treatment strategies in terms of inducing cell types

**Figure 4:**
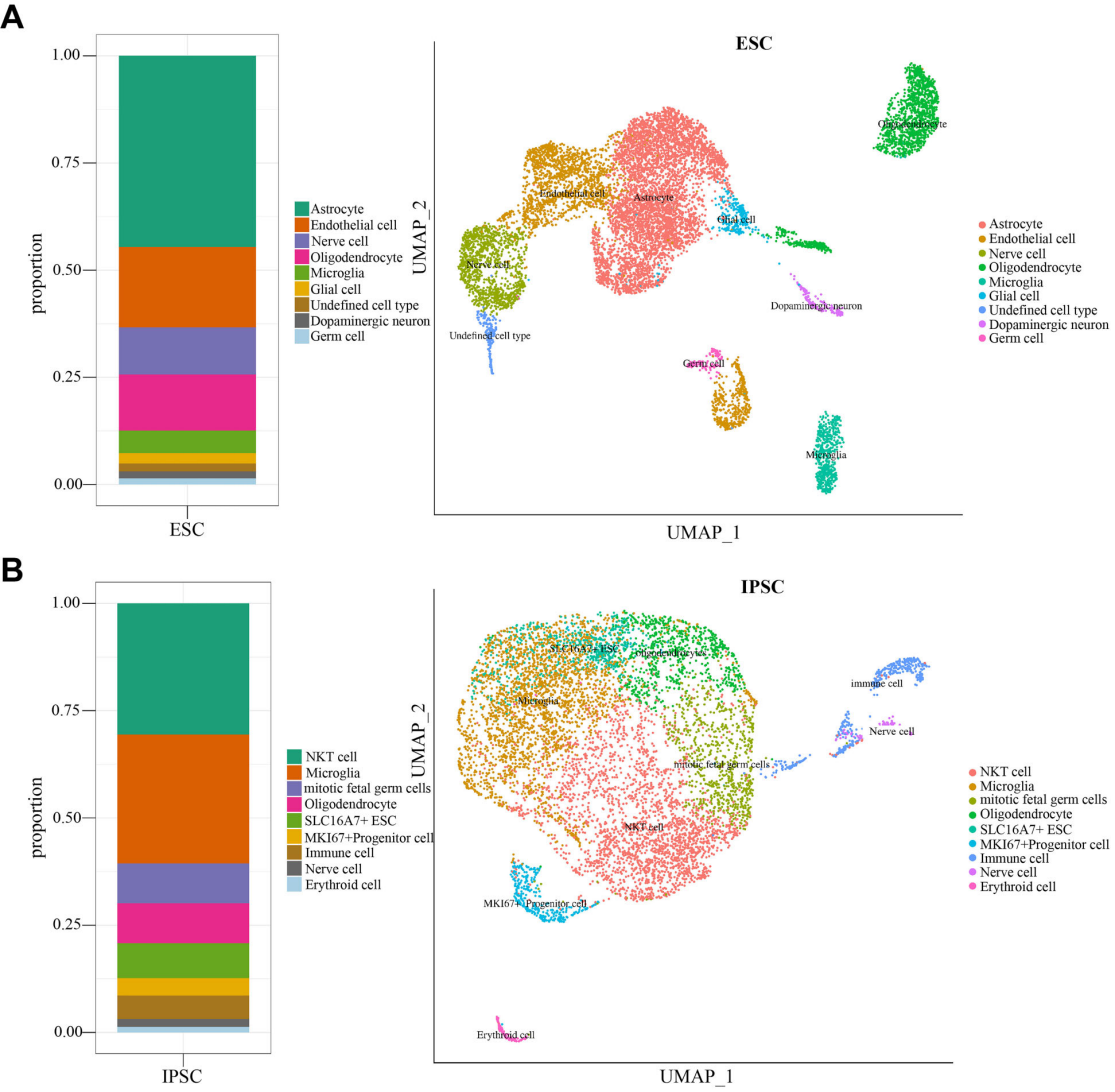
**A**, Based on existing research foundations and sample characteristics, the types of cell subgroups in the samples were estimated. Known gene markers corresponding to each cell subgroup were searched, and cell subtype classification and naming were performed based on known gene markers. The naming results are astrocytes, endothelial cells, neurons, oligodendrocytes, microglia, glial cells, undefined cell type, dopamine neurons, and germ cells. The cell type distribution map in embryonic stem cells (ESC) and the distribution of cell proportions in different groups are plotted based on these results, where the x-axis represents different groups and the y-axis represents the proportion of each cell type. **B**, Similarly, based on the naming method in ESC, the induced pluripotent stem cells (iPSC) naming results are NKT cells, microglia, dividing fetal germ cells, oligodendrocytes, SLC16A7+ ESCs, MKI67+ progenitor cells, immune cells, nerve cells, and erythroid cells. The cell type distribution map in iPSC and the distribution of cell proportions in different groups are plotted based on these results, where the x-axis represents different groups and the y-axis represents the proportion of each cell type.

To investigate the differences between iPSC and ESCs treatment strategies, cells were further clustered and classified. A total of 13 cell clusters were identified for iPSC and for ESCs, and cell names and expression patterns were analyzed (Figure 4A and B). Overall, the cell types transformed by both treatment strategies were similar, with the majority of cells transforming into glial cells and a small proportion of cells transforming into germ cells. Surprisingly, only the ESCs treatment strategy showed the presence of nerve cells and neurons (Figure 4A). Further exploration of the differences in cell types and quantities between the two treatment strategies revealed that, compared to the ESCs treatment strategy, the iPSC treatment strategy had a lower abundance of astrocytes and a higher quantity of NKT cells (natural killer T cell) (Figure 4B). In terms of shared cell types, iPSC showed a significantly higher expression of oligodendrocytes compared to ESCs. Additionally, iPSC treatment strategy lacked glial cells and endothelial cells, and especially also lacked dopaminergic neurons, which play a key role in ameliorating PD. These results suggested that the therapeutic effect of ESCs in PD may be achieved through a combination of different glial cells and nerve cells, while iPSC mainly shows a large number of immune cells on the surface.

### Differences in cell gene expression between iPSC and ESC treatment strategies

**Figure 5:**
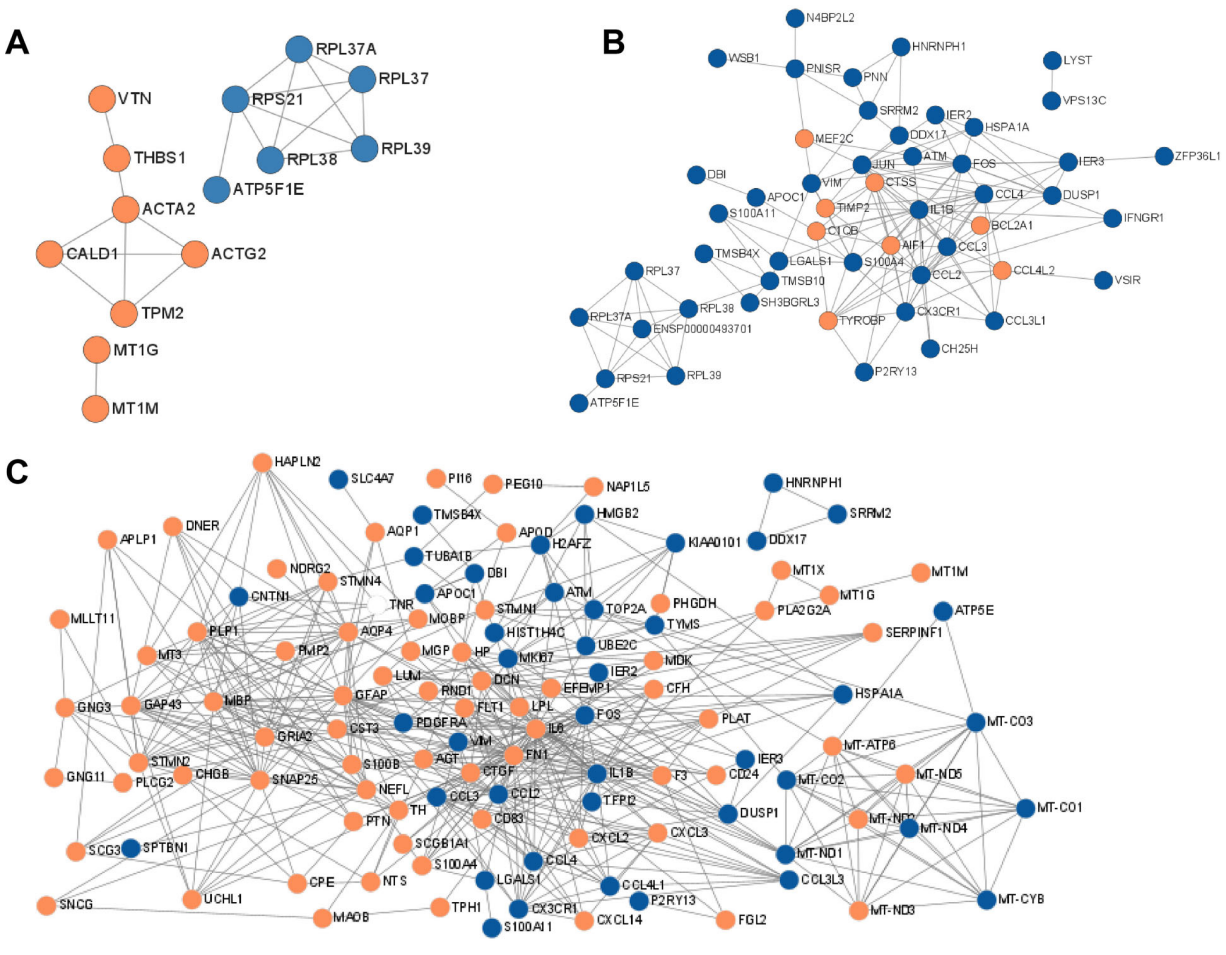
Marker genes of embryonic stem cells (ESCs) and induced pluripotent stem cells (iPSC) single-cell data were analyzed using STRING (https://string-db.org/). Based on the single-cell sequencing results of ESCs and iPSC, germ cells (**A**) and astrocytes (**B**) were identified in both. Therefore, a network graph of key genes between these two cell types and of ESC and iPSC cells, excluding germ and astrocytes cells, were plotted (**C**). The results showed that there was less interaction of key genes related to germ cells between iPSC and ESCs, while there was a close association of key genes between astrocytes and other cell types in both iPSC and ESCs.

Based on the STRING database and Cytoscape software, this study constructed gene interaction networks for the cell differentiation processes of iPSC and ESCs treatment strategies (Figure 5A-C). Firstly, in terms of shared cell types such as oligodendrocytes and germ cells, iPSC and ESCs did not show a strong correlation in germ cells during cell differentiation. However, in the case of oligodendrocytes, all genes involved in the cell differentiation process of ESCs showed a very strong correlation with the genes involved in iPSC cell differentiation. Among these genes, *AIF1* and *CTSS* showed the highest level of correlation, indicating that both treatment strategies may rely on a similar pathway in oligodendrocytes. Surprisingly, apart from the shared oligodendrocytes and germ cells, ESCs and iPSC showed a strong correlation in other genes, with a higher number of key genes in ESCs and a lower number in iPSC. Further analysis of the functions of these key genes revealed that iPSC mainly consisted of immune-related key genes, while ESCs had many key genes involved in neural growth and development, such as *TH* and *GAP43*. Thus, these results indicated a close association between iPSC and ESCs in improving PD, with ESCs having additional key genes related to neural development compared to iPSC, suggesting that iPSC mainly exerts its effects in improving PD through immune mechanisms.

### Differences in cell metabolism between iPSC and ESC treatment strategies

**Figure 6:**
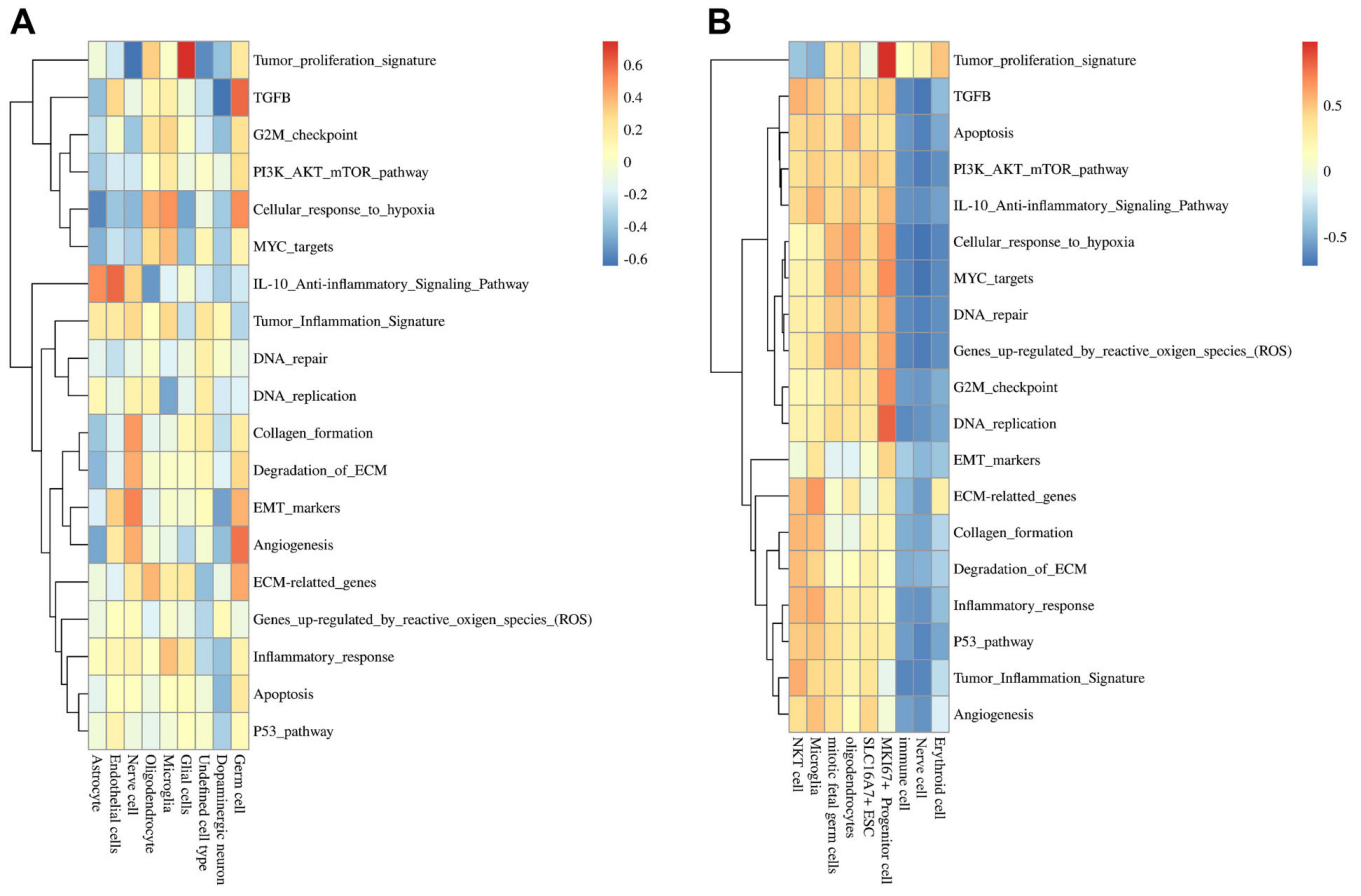
Metabolic analysis graphs comparing pathway scores between different groups of a certain cell population or between certain cell populations in some groups for embryonic stem cells (ESC) (**A**) and induced pluripotent stem cells (iPSC) (**B**). The results showed that the strongest reaction in ESCs was the tumor-proliferation signature of glial cells, and the weakest reactions were the tumor-proliferation signature of neurons and TGFB of dopaminergic neurons. In iPSC, the strongest reaction was the tumor-proliferation signature of MKI67+ progenitor cells, and the weakest reactions were TGFB and cellular response to hypoxia of nerve cells.

Based on the analysis of key genes, this study first calculated the average expression values of cells in iPSC and ESCs using the Average Expression function. Then, the GSVA package was used to calculate pathway scores for each cell subtype or group corresponding to the respective pathways. Finally, a heatmap was used for visualization to explore the differences in metabolism between iPSCs and ESCs. Overall, ESCs are involved in metabolic processes to varying degrees across different cell types, whereas for iPSCs, only erythroid cells, nerve cells, and immune cells are enriched at the metabolic level within the tumor-proliferation signature. Specifically, compared to ESCs, iPSCs exhibit a lesser inflammatory response and a more extensive involvement in DNA repair. However, iPSCs have an apoptotic process and are weaker in DNA repair compared to ESCs. In addition, the results showed that the strongest reaction in ESCs was the tumor-proliferation signature of glial cells, while the weakest reactions were the tumor-proliferation signature of nerve cells and the TGFB pathway of DA neurons (Figure 6A). In iPSCs, the strongest reaction was the tumor-proliferation signature of MKI67+ progenitor cells, while the weakest reactions were the TGFB pathway and the cellular response to hypoxia of nerve cells (Figure 6B). In terms of shared cell types, such as oligodendrocytes and germ cells, both iPSCs and ESCs showed similar reactions in oligodendrocytes, with the strongest reaction being the cellular response to hypoxia and the weakest reaction being DNA replication in ESCs and the G2M checkpoint in iPSCs. However, there were significant differences in reactions related to germ cells, with the strongest reaction in ESC being the TGFB pathway and the weakest reaction being the tumor inflammation signature. In iPSCs, the strongest reactions were MYC (c-Myc) targets and genes upregulated by reactive oxygen species, while the weakest reaction was EMT (epithelial to mesenchymal transition) markers. In summary, there were significant differences in the metabolic levels between iPSCs and ESCs, with iPSCs showing a weaker degree of enrichment in metabolic participation across different cell types compared to ESCs.

## Discussion

Based on the results of single-cell sequencing, we characterized the cell type profiles of ESC and iPSC treatment strategies and highlighted the richer cell types and enhanced neurotrophic promotion by ESCs in the treatment of PD. Moreover, our results revealed that iPSCs exhibited a lower inflammatory response at the metabolic level compared to ESCs, and we proposed that iPSCs should be induced more appropriately to fully exploit their potential for cell type enrichment, thereby better replace ESCs for the treatment of PD.

The current standard approach in the treatment of PD involves using l-DOPA as a precursor for DA synthesis to replenish depleted DA levels and achieve therapeutic goals. However, long-term research has shown that prolonged use of l-DOPA can lead to a serious motor complication, and this additional motor impairment can impact the quality of life of PD patients even more than the disease itself (18). Therefore, the discovery of ESCs and their potential to replenish damaged DA neurons rather than just supplementing DA makes them a very promising treatment strategy. ESCs can be indefinitely expanded to produce a large number of differentiated cells needed for transplantation and generally have fewer side effects compared to l-DOPA. However, the limitations of ESCs themselves have hindered the widespread application of this therapy. Fortunately, with the emergence and development of iPSC technology, current research has transitioned from initially restoring mouse fibroblasts to ESC-like iPSCs using four transcription factors (Oct4, Sox2, Klf4, and c-Myc) (19) to reprogramming human somatic cells into hiPSCs using similar transcription factors. Increasing experimental evidence suggests that iPSC-derived transplants are an ideal choice for the treatment of diseases related to ESCs (15,20,21). The reasons for this are as follows: 1) Single-cell sequencing results have shown that iPSCs produce fewer reactive astrocytes compared to ESCs, which can reduce neuroinflammation. Existing studies have demonstrated that reactive astrocytes can regulate microglial activation and microglia-induced neuroinflammation, leading to the death of neurons and oligodendrocytes (22,23); 2) Single-cell RNA-seq results of iPSCs did not detect 5-hydroxytryptamine neurons, indicating the safety of these transplants (10); 3) iPSCs do not have ethical and immunosuppressive restrictions. However, this study's experimental results indicated that iPSCs also have some limitations in pluripotency (10). For example, iPSCs lack certain neuronal development-related genes, such as *TH* (Tyrosine hydroxylase), which are present in ESCs. iPSCs also exhibit lower cellular diversity compared to ESCs. Therefore, the ideal choice would be to improve PD through the addition of certain inducing factors to make iPSCs more comprehensive and stable in differentiation.

Based on the previous single-cell sequencing results, iPSCs express excessive immune cells such as NKT cells and microglia, while lacking cells promoting neural development, such as neurons and astrocytes compared to ESCs. Therefore, to optimize the use of iPSCs for the treatment of PD, appropriate cues need to be added to guide their differentiation. Currently, numerous cues have been applied to induce iPSCs to differentiate into different cell types. For example, the combination of SB431542, an ALK (anaplastic lymphoma receptor tyrosine kinase) inhibitor, and Noggin can direct iPSCs to differentiate into neural progenitor cells (24). In addition, AICAR (acadesine/AICA nucleoside) and AMPK (adenosine 5'-monophosphate (AMP)-activated protein kinase) activators can induce the differentiation of neural stem cells into astrocytes (25). Furthermore, iPSCs in different states exhibit differences in proliferation and differentiation rates. Studies have shown that when cells from the same genomic background (nP002) are reprogrammed to a naive state, the cell doubling time of P002 is reduced from 36.1 h in the original iPSC state to 18.6 h (26). Therefore, converting iPSCs from their original state to a naive state can significantly accelerate cell proliferation, which should also be taken into account.

The main pathological pathway of PD is the substantia nigra-striatum pathway, characterized by the loss of DA neurons. Therefore, the key point of iPSC therapy is to regenerate healthy DA neurons *in vitro* using iPSCs and perform cell replacement transplantation to restore DA secretion levels in the brain, effectively alleviating PD symptoms. This is currently the most researched and clinically translated application of iPSCs in the field of neuroscience. Studies have shown that DA neurons derived from iPSCs have the potential to reconstruct neural networks. It is worth noting that PD, as a complex multisystem neurodegenerative disease, is not just characterized by the loss of DA neurons. There are also changes in other cell types in the brain, such as neuroinflammation caused by interactions between microglia and astrocytes, a significant reduction in oligodendrocytes in the PD pathological area (27), and even the gut microbiota (28-30), which is currently being studied as a potential origin of PD. Therefore, several studies have explored the use of iPSCs-cultured astrocytes and microglia to improve PD. In conclusion, these studies suggest that cell replacement therapy for PD should not be limited to DA neurons alone. It is essential to thoroughly analyze the underlying causes of the pathologic environment in PD and utilize the characteristics of iPSCs for a more comprehensive and effective treatment approach.

Currently, the clinical strategy for treating PD using iPSCs technology is advancing steadily, and several companies worldwide have been approved for clinical trials of iPSC-derived drugs targeting PD. Recent research has particularly achieved substantial improvements in most symptoms of PD patients using iPSCs grafts (31). In addition, studies on the application of personalized iPSC-derived DA progenitor cells in the treatment of PD have also shown varying degrees of efficacy (32). It is worth noting that we are still in the exploratory stage of iPSCs technology, and there remains uncertainty regarding the clinical readiness, applicability, and potential outcomes of this method. While some successes have been achieved, there have also been uncontrollable impacts, such as the propensity of iPSCs to cause teratoma formation in some cases (33,34). Furthermore, research has indicated the presence of mitochondrial defects, increased oxidative stress, lysosomal defects, and endolysosomal trafficking defects in neurons derived from iPSCs lines of PD patients 35). Although there are still many limitations to iPSCs, the latest research is continuously working to overcome these additional negative effects (17). It is believable that this technology has developed into a valuable tool for studying the pathogenesis of PD and for preclinical discovery and treatment evaluation. In the future, it is expected to bring about significant improvements for PD and possibly other diseases as well.
